# What is the best surgical approach for Pineal Region Tumors? A systematic literature review and anatomical comparative study

**DOI:** 10.1007/s00381-026-07336-3

**Published:** 2026-06-03

**Authors:** Davide Palombi, Ludovico Agostini, Riccardo Maria Brancaleone, Martina Offi, Edoardo Agosti, Simona Serioli, Barbara Buffoli, Rita Rezzani, Pierpaolo Mattogno, Lena Hirtler, Marco Maria Fontanella, Luca Massimi, Alessandro Olivi, Francesco Doglietto, Gianpiero Tamburrini

**Affiliations:** 1 Department of Pediatric Neurosurgery, Fondazione Policlinico Agostino Gemelli IRCCS, Università Cattolica del Sacro Cuore, 00168 Rome, Italy; 2 Department of Neuroscience, Università Cattolica del Sacro Cuore, 00168 Rome, Italy; 3 Department of Neurosurgery, Fondazione Policlinico Agostino Gemelli IRCCS, Università Cattolica del Sacro Cuore, 00168 Rome, Italy; 4 Department of Medical and Surgical Specialties, Radiological Sciences and Public Health, Neurosurgery Unit, University of Brescia, 25123 Brescia, Italy; 5 Section of Anatomy and Physiopathology, Department of Clinical and Experimental Sciences, University of Brescia, 25123 Brescia, Italy; 6 Division of Anatomy, Center for Anatomy and Cell Biology, Medical University of Vienna, Vienna, Austria

**Keywords:** Pineal gland, Pineal region tumors, Surgical approaches

## Abstract

**Purpose:**

Pineal region tumors pose significant surgical challenges due to their deep location and proximity to critical neurovascular structures. Despite multiple described techniques, no consensus exists on the optimal surgical approach, with the supracerebellar infratentorial (SCIT) and occipital interhemispheric transtentorial (OITA) approaches representing the most adopted corridors.

**Methods:**

A systematic literature review was conducted to evaluate surgical approaches for pineal region tumors and their associated complication profiles. In addition, anatomical dissections combined with a tumor simulation technique were used as illustrative tools to compare exposure patterns obtained through SCIT, SCIT with tentorial incision (SCIT + T), and OITA approaches.

**Results:**

The systematic review, comprising 27 studies and 1774 patients, identified OITA (42.21%) and SCIT (38.27%) as the most frequently employed approaches. SCIT was more commonly associated with cerebellar complications, cerebrospinal fluid leaks, meningitis, and Parinaud’s syndrome (*p* < 0.005), whereas OITA was associated with a higher incidence of homonymous hemianopia (*p* = 0.0011).

From an anatomical perspective, infratentorial approaches provided broader exposure of the inferior quadrants (Q1, Q2), while OITA offered greater visualization of the superior ipsilateral quadrant (Q3). The addition of a tentorial incision to SCIT facilitated visualization of supratentorial quadrants (Q3, Q4), which are otherwise less accessible through the standard infratentorial corridor. Tumor simulations further illustrated that approach selection is influenced by the tumor’s relationship to the deep venous system, with supratentorial routes favoring lesions anterior to the veins and infratentorial corridors favoring posterior extension.

**Conclusions:**

Selection between SCIT and OITA should be individualized based on tumor characteristics, venous relationships, and tentorial anatomy. Tentorial incision may expand the versatility of SCIT in selected cases.

## Introduction

Pineal region tumors present unique challenges due to their deep location within the brain near critical neurovascular structures [1–3]. The pineal gland is situated at the junction of the midbrain and thalamus, making access to tumors complex and demanding [4].

Surgical intervention in pineal region tumors is necessary for diagnosis, symptom relief, and potential resection [5, 6]. However, due to the anatomical complexity of this region, no consensus exists on the optimal surgical approach [7–10]. The two main corridors used are the supracerebellar infratentorial (SCIT) and the occipital interhemispheric transtentorial (OITA) approaches, each with distinct advantages and limitations [1, 11–14].

Initially described by Poppen and refined by Jamieson, the OITA approach provides excellent lateral exposure and is well-suited for steep tentorium cases [11, 15, 16]. However, it is limited by obstructions at the torcular-transverse sinus junction and at the confluence of the internal cerebral veins (ICVs) with the vein of Galen (VoG), potentially restricting visualization and leading to incomplete resection [17]. The SCIT approach, initially developed by Krause and later refined by Stein, is widely used in the treatment of pineal tumors [2]. It benefits from a natural cerebellar descent in the sitting position, enhancing the surgical field. However, it is less suitable for patients with a steep tentorium and carries risks, such as venous air embolism and cerebellar infarction due to bridging-vein sacrifice [13, 17].

Over the years, microsurgical approaches to the pineal region have evolved through extensive anatomical investigation and clinical experience [13, 18–22]. Detailed anatomical studies emphasized the importance of tentorial configuration, venous anatomy, and microsurgical corridors in determining safe access to the pineal region [13, 16, 21, 22]. Similarly, many authors highlighted that approach selection should not rely on a single anatomical parameter, but rather on a balanced evaluation of exposure, venous preservation, and patient-specific factors [8, 17, 18, 20, 23, 24]. Despite multiple studies, no definitive criteria exist to determine the optimal approach, and no general agreement has been established [8, 17].

We performed a systematic literature review to evaluate the most widely employed surgical approaches and assess potential differences in complication rates. In addition, anatomical tumor simulation was used as an illustrative tool to support and contextualize the review's findings, analyzing and comparing the exposure provided by SCIT and OITA, including the potential role of a tentorial incision during the supracerebellar approach and using a tumor simulation technique, as suggested by the article by Oyama et al. [25].

Ultimately, this study aims to contribute to a more nuanced and individualized surgical decision-making process, in which anatomical configuration, tumor extension, venous relationships, and patient-specific factors are considered when selecting the most appropriate approach for pineal region tumors.

## Materials & methods

### Systematic literature review

#### Study design and protocol

The review followed the Preferred Reporting Items for Systematic Reviews and Meta-Analyses (PRISMA) guidelines [26]. The systematic review was registered with PROSPERO under the protocol number CRD420251059155.

#### Search strategy

A literature search was conducted using PubMed, Medline, Cochrane, Embase, Scopus, and the Web of Science search engine, encompassing the period from January 1984 to January 2025, utilizing MeSH headings of “Pineal gland,” “Pineal tumor,” “Pineal region,” and “approach” or “surgery.” A filter for “humans” and the “English” language was applied during the search. The abstracts of all manuscripts were reviewed, and relevant articles were identified.

#### Eligibility criteria

The inclusion criteria were patients who underwent surgery for tumors in the pineal region, including those of any age group (both pediatric and adult populations), to maximize the breadth of the evidence base. Eligible study designs include retrospective cohort studies and comparative studies. Exclusion criteria include patients who underwent biopsy only. Studies that do not report the primary outcome of interest or contain incomplete data were excluded. Furthermore, case reports, case series with fewer than 20 patients, editorials, and review articles were excluded from analysis. The primary outcome assessed is the choice of the approach. The secondary outcome was the complication rate between the two approaches.

#### Study selection and data collection

Two independent reviewers (R.M.B. and L.A) screened each article in two stages. Initially, they screened article titles and abstracts, followed by a full-text review of the selected articles. Any disagreements were resolved by consulting with a third senior reviewer (L.M). Patient demographics, surgical approaches, histology, and complication rates were extracted from each included study (Table 1).

**Table 1:** Summary of study characteristics, patient demographics, surgical approaches, histology, and reported complications across the included studies

Authors	Year	N	Age (y)	Sex	Biopsy alone	Surgery	Approach	Histology	Complications	EOR (GTR/STR/PR)
Hoffman et al. [27]	1984	61	0–14	40 M (66%) 21 F (34%)	0	40	1 OITA (3%) 2 SCIT (5%) 37 Other (31 TC, 6TV- 93%) 21 no procedure	24 GCT (39%) 14 PPT (23%) 8 Glioma (13%) 15 Unverified (25%)	Death (8, 20%)	N/A
Luo et al. [15]	1989	64	2–43	50 M (82%) 14 F (18%)	0	64	64 OITA (100%) 0 SCIT (0%) 0 Other (0%)	Germinoma (28, 43%) Teratoma (14, 21%) Glioma (12, 18%) PPT (4, 6%) Dermoid/epidermoid (4, 6%) Other (2, 3%)	Death (2, 3%)	N/A
Pluchino et al. [28]	1989	40	22 (4–56)	26 M (65%) 14 F (35%)	15	25	25 OITA (68%) 0 SCIT (0%) 15 Other (SB-32%)	12 GCT (30%) 10 PPT (25%) 10 Glioma (25%) 8 Other (20%)	Death (2, 8%) Parinaud’s Syndrome (n.a.)	N/A
Bruce et al. [2]	1995	154	29(4–69)	102 M (66%) 52 F (33%)	0	160	0 OITA (0%) 137 SCIT (89%) 23 Other (ST-11%)	GCT (57, 37%) Glioma (43, 28%) PPT (35, 23%) Meningioma (9, 6%) Other (10, 6%)	Death (6, 4%) EOM dysfunction (70, 45%) Ataxia (31,14%) Hemorrhage (10, 7%) Extrapyramidal syndrome (5, 3%) Hemiparesis (5, 3%) Homonimous Hemianopia (3, 2%) Others (4, 3%)	GTR: 45%
Shin et al. [29]	1998	25	9.5(14 m-15)	18 M (72%) 7 F (28%)	1	24	21 OITA (84%) 1 SCIT (4%) 3 Others( 2 TCTV, 1 SB-12%)	GCT (14, 56%) PPT (6, 24%) Glioma (3, 12%) Cavernous Angioma (2, 6%)	Homonimous hemianopia (7, 28%) Parinaud’s Syndrome (6, 24%) Forced lateral gaze (4, 16%) CN VI palsy (3, 12%) Seizures (5, 20%) Hydrocephalus (3, 12%)	GTR: 62%, STR: 29%, PR: 9%
Drummond et al. [30]	1999	37	9.6 (0–16)	31 M (84%) 6 F (16%)	13	21	0 OITA (0%) 4 SCIT (11%) 30 Others (9 TC, 5 ST-SO, 3 ST-TC, 13 SB) 3 no procedure	Germinoma (13, 35%) GCT (3, 8%) Yolk sac-tumor (2, 5%) Teratoma (2, 5%) PPT (8, 22%) Glioma (3, 8%) Unverified (6, 16%)	Death (2, 5%) Neurological worsening (6, 16%) CSF leak (1, 3%) Wound infection (1, 3%) Seizures (2, 5%)	GTR: 19%, STR: 81%
Konovalov et al. [3]	2003	287	20 (3–69)	161 M (56%) 126 (44%)	43	244	138 OITA (54%) 87 SCIT(34%) 62 Other (4 Subchoroidal, 4 TV, 21 Combine, 33 NA%)	GCT (87, 31%) PPT (75, 27%) Glioma (77, 27%) Other (43, 15%)	Intralesional haemorrhage (29, 11%) Sub/epidural hematoma (9, 4%) Meningitis (15, 6%) Parinaud’s syndrome76, 31%) Homonimous hemianopia (12, 4%) Death (20, 7%)	GTR: 58%, STR: 29%, PR: 13%
Hernesniemi et al. [31]	2008	119	34(0–78)	51 M(43%) 68 F (57%)	0	119	8 OITA (7%) 111 SCIT (93%) 0 Other (0%)	GCT (13, 11%) Pinealoblastoma (10, 8%) Pineocytoma (22, 18%) Glioma (26, 22%) Meningioma (10, 8%) Galenic vein malformations (6, 6%) AVM (2, 2%) Cavernoma (4, 4%)	Epidural hematoma (1, 1%) Parinaud’s syndrome (9, 8%) Meningitis (2, 2%) CSF fistula (1, 1%) Wound infection (3, 3%) Memory impairment (2, 2%) Hemiparesis (2, 2%) Trochlear/abducens palsy (2, 2%) Shunt infection (1, 1%)	GTR: 88%, STR: 12%
Sajko et al. [9]	2009	39	24(4–66)	18 M (46%) 21 F (54%)	0	39	0 OITA (0%) 39 SCIT (100%) 0 Other (0%)	Pineocytoma (13, 33%) Germinoma (10, 26%) Pineal cyst (7, 18%) Pinealoblastoma (3, 8%) Pilocytic astrocytoma (2, 5%) Other (4, 10%)	Seizures (3, 8%) Meningitis (4, 10%) CSF leak (3, 8%) Hemiparesis (2, 5%) Diabetes insipidus (1, 3%)	N/A
Davidson et al. [32]	2011	26	7 (7 m-17)	11 M (42%) 15 F (58%)	2	27	0 OITA (0%) 0 SCIT (0%) 29 Other (PIRA-100%)	PNET (7–27%) ATRT (4–15%) Astrocytoma (5–19%) Choroid plexus carcinoma (2, 8%) GCT (2, 8%) Trigeminal schwannoma (2, 8%) Other (4, 15%)	Homonimous hemianopia (2–8%) Hemiparesis (3–12%) Parinaud’s syndrome (2, 8%) Facial nerve palsy (1, 4%) Infection (1, 4%)	GTR: 74%, STR: 19%, PR: 7%
Kodera et al. [33]	2011	26	31.6 (9–61)	15 M (58%) 11 (42%)	0	26	0 OITA (0%) 26 SCIT (100%) 0 Other (0%)	Pineocytoma (10, 38%) Astrocitoma (6, 23%) Pineal cyst (4, 15%) Malignant glioma (2, 8%) Other (4, 15%)	Parinaud’s syndrome (1, 4%) Diplopia (1, 4%)	GTR: 88.5%, STR: 11.5%
Oliveira et al. [34]	2013	32	27,2 (4–65)	21 M (66%) 11 F (33%)	0	32	0 OITA (0%) 32 SCIT (100%) 0 Other (0%)	Germinoma (n.3, 9%) Teratoma (n.3, 9%) Pinealoblastoma (n.6, 19%) Low grade Astrocitoma (n. 6, 19%), GBM (n.2,6%) Metastasis (n.2, 6%) Ependymoma (n.1, 3%) Epidermoid tumor (n.1, 3%) Cavernoma (n.1, 3%) Arachnoid cyst (n.2, 6%) PTID (n.2, 6%)	Parinaud’s syndrome (n.14, 44%) Ataxia (n.3, 9%) CSF fisula (n.1, 3%)	N/A
Qiu et al. [35]	2014	15	51,3(34–61)	5 M (33%)10 F (67%)	0	15	15 OITA (100%) 0 SCIT (0%) 0 Other (0%)	Meningioma (15, 100%)	Homonimous hemianopia (2, 13%) Parinaud Syndrome (1, 7%) Diplopia (1, 7%)	GTR: 73.3%, NTR: 20%, STR: 6.7%
Mottolese et al. [36]	2015	232	39 (8 m, 74)	n.a	0	232	201 OITA (87%) 31 SCIT (13%) 0 Other (0%)	Pinealocitoma (6, 19%) Papillary tumor (3, 10%) Germinoma (7, 23%) Teratoma (2, 6%) Pineal cyst (4, 13%) Glioma (9, 29%)	Diffuse cerebellar oedema (1, 3%) IV NC palsy (1, 3%) Parinaud’s syndrome (2, 6%) Gait ataxia (2, 6%)	N/A
Kotwica et al. [37]	2017	22	44 (21–62)	n.a	0	22	0 OITA (0%) 22 SCIT (100%) 0 Other (0%)	Pineocytoma (7, 32%) Pineoblastoma (3, 14%) PNET (6, 27%) GBM (2, 9%) Metastasis (2, 9%) Cavernoma (1, 5%) Central neurinoma (1, 5%)	None	N/A
Abecassis et al. [38]	2017	50	25 (6 m, 66)	30 M (60%), 20 F (40%)	13	37	4 OITA (8%) 16 SCIT (32%) 30 Other ( 13 TCTFC, 13 ETV, 4 ATC-30%)	GCT (19, 38%) PPT (14, 28%) Papillary tumor (5, 10%) Pylocitic astrocytoma (4, 8%) Meningioma (3, 6%) Teratoma (3, 6%) ATRT (1, 2%) GBM (1, 2%) Others (3, 6%)	Death (3, 6%) Parinaud’s syndrome (14, 28%) CSF Leak (3, 6%) Other (8, 16%)	GTR: 62%
Rosenberg et al. [14]	2019	60	36,4(16–75)	30 M (50%) 30 F (30%)	0	60	13 OITA (21%) 47 SCIT (79%) 0 Other (0%)	N.S	Respiratory (5, 8%) Hematological (4, 7%) Cardiovascular (2, 3%) Deep Surgical Site Infection (1, 2%)	N/A
Choque-Velazquez et al. [39]	2020	112	30,0 (19,8–40,5)	41 M (37%) 71 F (64%)	0	112	0 OITA (0%) 112 SCIT ( 55 m-SCIT 57 p-SCIT-100%) 0 Other (0%)	GCT (11, 10%) Glioma (14, 13%) Meningioma 4, 4%) PPT (19, 17%) Pineal Cyst (57, 51%) Other (7, 6%)	CSF leak (4, 4%) Meningitis (9, 8%) Wound infection (6, 6%) Pseudomeningocele (5, 5%) Other (12,11%)	GTR: 87.5%, STR: 12.5%
Richards et al. [40]	2021	38	(3–71)	27 M (71%) 11 F (29%)	17	21	10 OITA (26%) 8 SCIT (21%) 20 Other ( 17 ETV, 3 SB—53%)	GCT (9, 24%) PPT (13, 34%) Glioma (5, 13%) Meningioma (2, 5%) Metastasis (2, 5%) Teratoma (2, 5%) Others (5, 13%)	Hemorrhage (2, 6%) Occipital lobe ischemia (4, 11%)	SCIT: ~ 97%, OTT: ~ 60%
Hu et al. [41]	2022	72	23,5 (10 m, 70)	54 M (75%) 18 F (25%)	0	72	10 OITA (14%) 46 SCIT(64%) 16 Other (TCTFC- 22%)	GCT (24, 33%) Teratoma (11, 15%) Glioma (15, 21%) Meningioma (6, 8%) PPT (11, 15%) Cholesteatoma (2, 3%) Hemangioma (2, 3%) Choriocarcinoma (1, 1%)	Death (1, 1%) Coma (1, 1%) Ataxia (2, 2%) Parinaud’s syndrome (9, 13%) Subcutaneous/subdural effusion (2, 3%)	GTR: 88.9%, STR: 11.1%
Tomita et al. [10]	2023	92	10,3 (3 m-21)	63 M (68%) 17 F (18%) NS:1 2(13%)	2	90	80 OITA (87%) 5 SCIT (5%) 7 Other ( 3 IE-TC, 4 TV- 8%)	GCT (n.32, 35%) Benign gliomas (n.22, 24%) PPT (n.13, 14%) ATRT (n.5, 5%) Papillary tumor (n.3, 3%) Medulloblastoma (n.2, 2%) Epydermoid cyst (n.2, 2%) GBM (n.1, 1%) N.S. (n.12, 13%)	Death (1, 1%) Parinaud’s syndrome(24, 26%) Hemiparesis (2, 2%) Ataxia (2, 2%) Subdural collection (3, 3%) Surgical cave bleeding (1, 1%)	GTR: 68.75%, STR: 16.25%, PR: 12.5%
Bozkurt et al. [5]	2023	27	36(18–66)	14 M (52%) 13 F (48)	0	27	27 OITA (POita-100%) 0 SCIT (0%) 0 Other (0%)	Pineal cell tumor (15, 55%) GCT (4, 15%) Glioma (4, 15%) Other (4, 15%)	Parinaud syndrome (2, 8%) Acute hydrocephalus (1, 4%) Mild hemiparesis (1, 4%)	GTR: 96.3%, STR: 3.7%
Patel et al. [42]	2024	43	10 (5 m-22)	22 M (51%) 21 F (49%)	17	26	0 OITA (0%) 11 SCIT (25%) 32 Other (19 PIH, 13 Other- 75%)	Germinoma (13, 30%) PPT (10, 23%) Glioma (6, 14%) Teratoma (4, 9%) ATRT (2, 5%) Other (8, 21%)	Infection (2, 5%) Parinaud’s syndrome (3, 7%) Homonimous hemianopia (2%) Left-side hyposthenia (1, 2%) Fixed pupil (1, 2%) Pseudomeningocele (1, 2%)	GTR: 50%
Milisavljevic et al. [8]	2024	69	26(4 m, 67)	37 M (54%) 32 F (46%)	0	69	28 OITA (41%%) 41 SCIT (59%) 0 Other (0%)	N.S	Air embolism (7% SCIT group) Gait ataxia (12% SCIT group) Homonimous hemianopia (20% OITA group) Parinaud’s syndrome (7%) CNS infection (1%)	SCIT: 78%, OTT: 50%
Ahmed et al. [1]	2025	32	39(12–75)	13 M (41%) 19F(59%)	0	32	0 OITA (0%) 32 SCIT (100%) 0 Other (0%)	Pineal cyst (12, 38%) PPT (9, 28%) Metastasis (3, 9%) Astrocitoma (2, 6%)	Temporary CN palsy (4, 13%) CSF leak (3, 9%) Hemorrhage (2, 6%) Venous thalamic infarction (1, 3%)	GTR: 72%, STR: 28%
Total		1774			123	1660	SCIT (632) OITA (697) Other (304) 1802			

*N.A.* Not available, *N.S.* Not Specified, *IE-TC* Inter-Emispheric Trans-Callosal, *TV* Trans-Vermian, *GCT* Germ-Cells Tumor, *ATRT* Atypical Theratoid-Rhabdoid Tumor, *Tctfc* Transcallosal-lateral ventricle-choroid fissure approach, *EVD* External ventricular drainage, *ETV* Endoscopic third ventriculostomy, *ATC* ANterior Trans-Choroisal, *m-SCIT* midline supra-cerebellar Infra-Tentorial, *p-SCIT* paramedian Supra Cerebellar Infra Tentorial, *PPT* Pineal Parenchimal Tumor (PIH- posterior interhemispheric trans-callosal), *TCTV* Transcortical transventricular, *TC* Trans-Callosal, *ST-SO* Supra-Tentorial sub-occipital, *ST-TC* supra-tentorial trans-cortical, *SB* Stereotactic biopsy, *GTR* gross-total resection, *STR* sub-total resection

#### Risk of bias assessment

The risk of bias in the studies included in this systematic review was assessed using the JBI Critical Appraisal tools for observational studies and case series [43]. Two reviewers (R.M.B and L.A) independently evaluated the study quality, resolving discrepancies through discussion. A third senior reviewer (L.M) arbitrated any unresolved disagreements. Table 2 provides a detailed description of the bias evaluation risk.

**Table 2:** Risk of bias assessment of the included studies according to the JBI Critical Appraisal Tool

Author										
Hoffman et al. [27]										
Luo et al. [15]										
Pluchino et al. [28]										
Bruce et al. [2]										
Shin et al. [29]										
Drummond et al. [30]										
Konovalov et al. [3]										
Hernesniemi et al. [31]										
Sajko et al. [9]										
Davidson et al. [32]										
Kodera et al. [33]										
Oliveira et al. [34]										
Qiu et al. [35]										
Mottolese et al. [36]										
Kotwica et al. [37]										
Abecassis et al. [38]										
Rosenberg et al. [14]										
Choque-Velazquez et al. [39]										
Richards et al. [40]										
Hu et al. [41]										
Tomita et al. [10]										
Bozkurt et al. [5]										
Patel et al. [42]										
Milisavljevic et al. [8]										
Ahmed et al. [1]										

#### Data synthesis

Complication rates between the SCIT and the OITA, as found in the literature, were compared using Chi-square tests. The tumor exposure and surgical volume of the two different approaches were compared. The Mann–Whitney U test was used to compare the three approaches within each quadrant. SPSS Statistics 25.0 v (IBM Corp., Armonk, NY, USA) was used for statistical analysis. A p-value < 0.05 was defined as significant.

### Comparative study

#### Preparation of specimens and neuronavigation

A total of 2 formalin-fixed specimens were dissected. In the injected specimen, the arterial system was injected with red silicone and the venous system with blue silicone to enhance vascular identification. The second specimen was not injected, as it was used solely for comparative quantitative assessment with a less steep tentorial angle, without the intent of vascular visualization. Each specimen underwent a 128-slice multidetector computed tomography scan (Somatom Definition Flash, Siemens, Forcheim, Germany). Subsequently, the Digital Imaging and Communications in Medicine (DICOM) records of the CT scans were transferred to a specialized neuronavigation software program (v. 1, GTx-Eyes II Approach Viewer, University Health Network, University of Toronto, Toronto, ON, Canada).

#### Surgical approaches to dissection

The dissections were conducted at the Anatomy Laboratory of the University of Brescia (Italy) using conventional microsurgical and endoscopic tools from Karl Storz® (Tüttlingen, Germany). To capture and record anatomical details, a Leica M320® surgical microscope (Leica Microsystems Srl, Buccinasco, Italy) and a 4 K camera head from Olympus® (Segrate, Italy) were employed.

For each specimen, three sequential surgical steps were performed to simulate tumor resection and analyze surgical exposure in a step-by-step way:

**Fig. 1:**
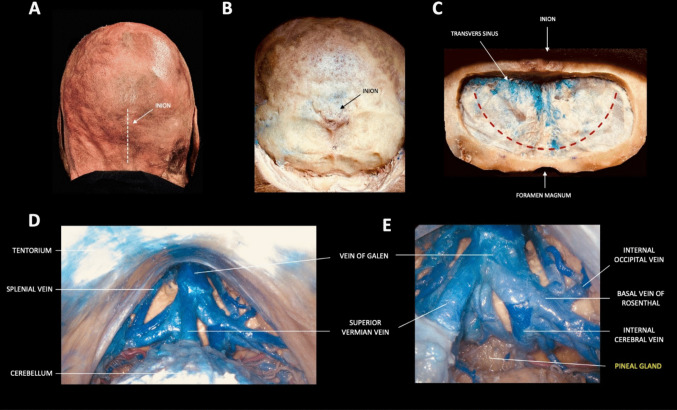
**A**–**E**. Step-by-step dissection illustrating the supracerebellar infratentorial (SCIT) approach

**Fig. 2:**
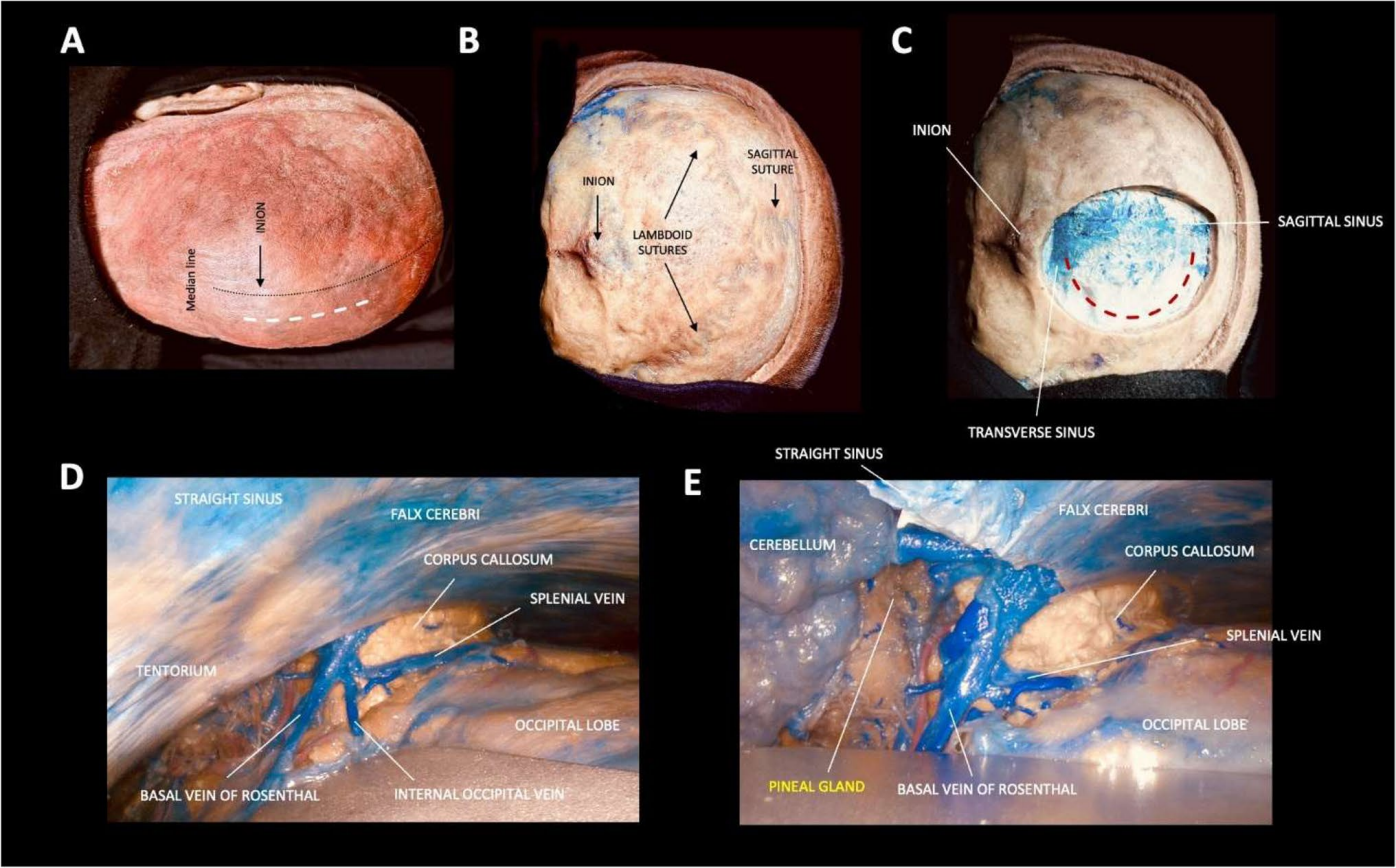
**A**–**E**. Step-by-step dissection illustrating the occipital interhemispheric transtentorial (OITA) approach

**Fig. 3:**
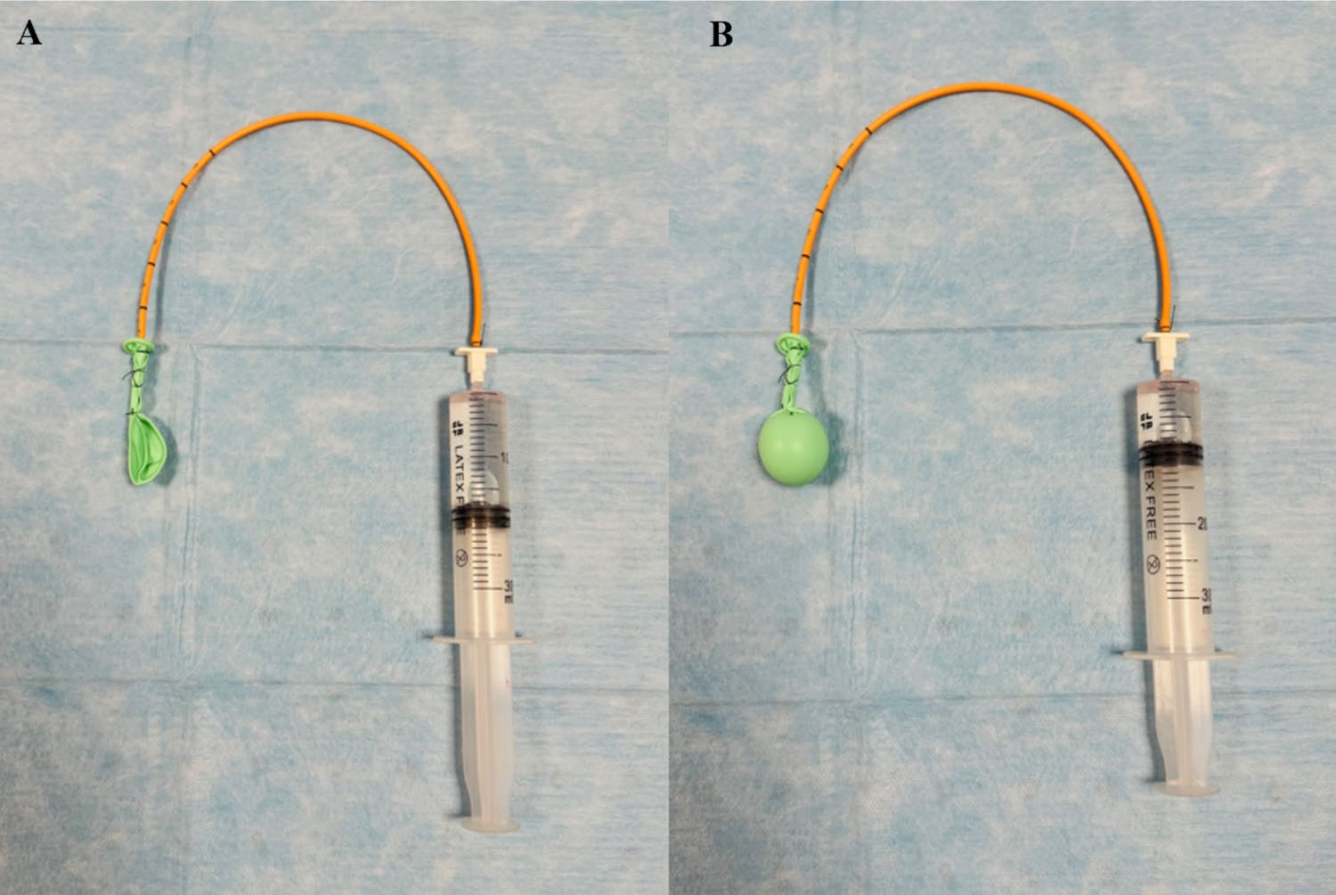
Illustration of the water-balloon technique simulating a pineal-region tumor. The latex balloon attached to a ventricular catheter is shown deflated (**A**) and inflated with water and CT-contrast agent to reproduce a pineal-region mass (**B**)

**Fig. 4:**
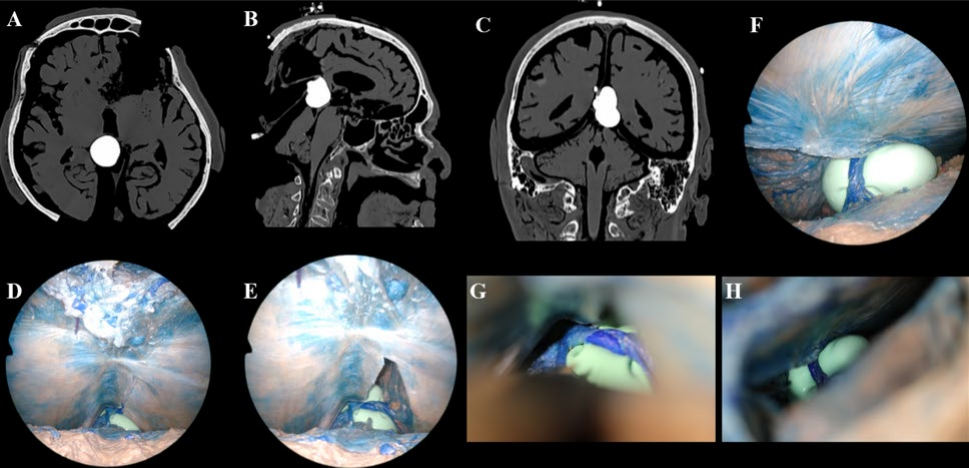
**A**–**H**. Tumor simulation in Head 1 demonstrating exposure with the SCIT and OITA approaches

**Fig. 5:**
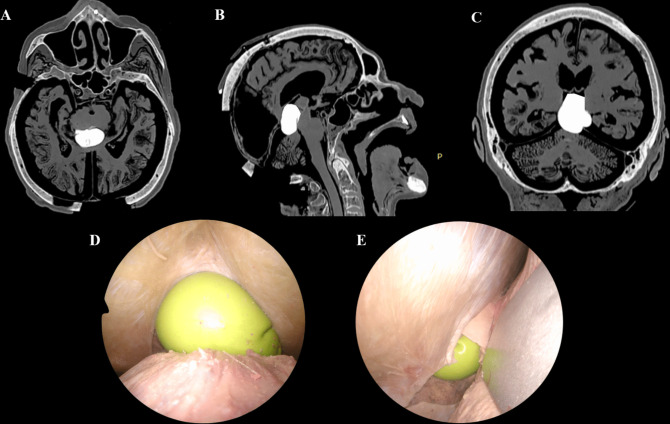
**A**–**E**. Tumor simulation in Head 2 demonstrating exposure with the SCIT and OITA approaches

1. The SCIT approach was performed first to assess the exposure area provided by its corridor. The head is positioned in a semi-sitting position, with neck flexion [2]. Tentorial opening was then performed at the end of the approach (SCIT + T). (Fig. 1A-E)
2. The OITA approach was then performed. The head was positioned in the prone position and rotated 15–30 degrees toward the operating side. It was anteflexed 15 degrees, simulating the actual surgical approach [11]. (Fig. 2A-E)

#### Tumor simulation

A balloon was inserted via the contralateral OITA approach for the OITA simulation and SCIT/SCIT + T simulation and connected to a 30-ml syringe to infuse different volumes of water and CT-contrast agent and was anchored to a ventricular catheter (Fig. 3A-B). After visual confirmation of its correct positioning at the level of the pineal gland, the balloon was inflated and filled with a CT contrast medium to simulate the tumor and the vein’s displacement. This “water balloon tumor” (WBT) mimicked a solid tumor, similar to those commonly seen in patients with pineal-region tumors. (Fig. 4A-H and 5A-E) To test and compare the visibility of surrounding structures using the two approaches, two volumes were used to inflate the water balloons: 10 mL and 15 mL for Tumors 1 and 2, respectively. Tumor 1 was paramedian and anterior to the venous complex, while Tumor 2 was a median and posterior one.

#### Volume quantification and comparison

Quantifications were obtained with an optical neuronavigation system (Polaris Vicra®, NDI, Waterloo, Canada) coupled with GTx-Eyes II software (ApproachViewer, part of GTx-Eyes II, University Health Network, Toronto, Canada). Each surgical approach was quantified 10 times per specimen, and the surgical corridors were quantitatively assessed using neuronavigation-based anatomical methodologies previously validated and applied in comparative neurosurgical studies, where surgical exposure accounted for the full working volume achievable when instruments are angled across the midline of the surgical corridor, thereby reflecting the maximal reachable area beyond the direct line of sight of the approach [44–46].

To facilitate interpretation, the surgical exposure around the pineal region was divided into four anatomical quadrants defined by two perpendicular planes: the tentorium cerebelli (separating supratentorial from infratentorial compartments) and the interhemispheric midline (separating left from right). The resulting quadrants were: Q1 (infratentorial–ipsilateral to the OITA entry point), Q2 (infratentorial–contralateral), Q3 (supratentorial–ipsilateral), and Q4 (supratentorial–contralateral). Statistical comparisons of surgical exposure volumes across approaches were performed using the Mann–Whitney U test (significance threshold: *p* < 0.05), and a HeatMap was performed to provide a visual summary of the exposure patterns across quadrants and approaches for both specimens, with color intensity reflecting the relative degree of exposure achieved (blue indicates low surgical exposure and red indicates high surgical exposure).

## Results

### Systematic literature review

#### Study selection

**Fig. 6:**
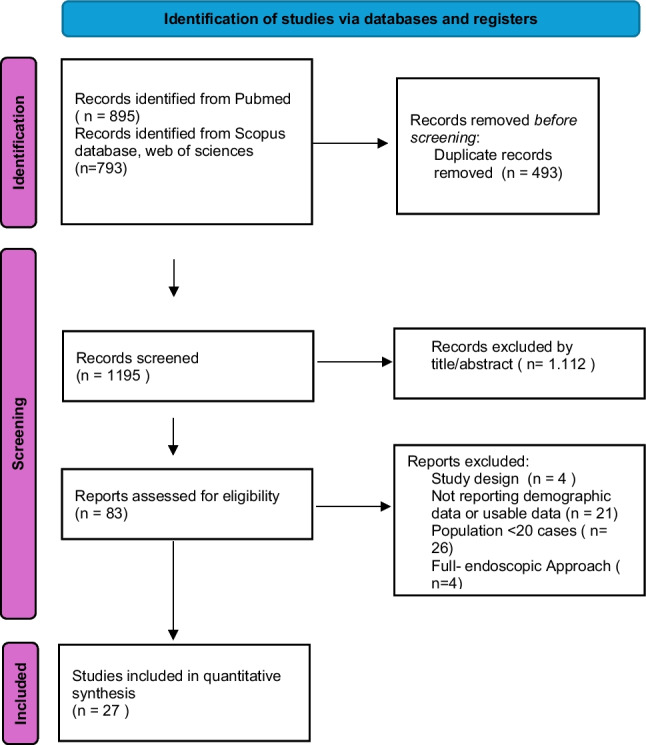
PRISMA flow diagram summarizing the study selection process

The initial database search identified 1688 records. A total of 27 studies met the inclusion criteria and were included in the study. The PRISMA flow diagram details the study selection process (Fig. 6). A total of 1774 patients were included in all reviewed studies. Of these, 123 patients underwent biopsy alone, while 1651 underwent surgical resection.

#### Study characteristics

The included studies ranged from 1984 to 2025 and included pediatric and adult populations. The data included retrospective case series with variable levels of clinical detail, as well as all reported surgical outcomes and complications associated with pineal region tumor surgery. All included studies were retrospective and classified as LOE 3.

#### Risk of bias within studies

Using the JBI Critical Appraisal Tool, most studies showed moderate to good methodological quality [43]. Older reports often lacked clarity in patient selection, confounder management, and outcome reporting, indicating a higher risk of bias [2, 3, 15, 27]. In contrast, more recent studies fulfilled most criteria, reflecting improved methodological standards [1, 8, 10, 41]. Overall, the risk of bias was heterogeneous, ranging from high in early retrospective series to low in recent cohort studies.

#### Approach’s choices, complications summary and extent of resection

Table 1 summarizes the study’s variables and data. Surgical approaches included the SCIT approach in 632 cases (38.27%), the OITA approach in 697 cases (42.21%), and other surgical techniques in 304 cases (18.41%). The remaining 18.41% of cases were performed using a heterogeneous group of techniques, including transcallosal, transventricular, endoscopic, and combined approaches.

Among complications reported in the OITA and SCIT cohorts, Parinaud’s syndrome was the most frequently reported complication, affecting 178 patients (10.78%). Other notable complications included death in 63 cases (3.81%), ataxia in 43 cases (2.6%), homonymous hemianopia in 41 cases (2.48%), and meningitis in 36 cases (2.18%). Cerebrospinal fluid (CSF) leak occurred in 27 patients (1.64%), and hemorrhage was reported in 22 cases (1.34%). Additional complications were less frequent but clinically relevant, including wound infections (1.28%), seizures (0.91%), pseudomeningocele (0.37%), and air embolism (0.5). Rare complications included coma, venous infarction, and death, each occurring in less than 0.1% of the cohort.

The extent of resection (EOR) data were extractable from 18 of the 27 included studies; the remaining studies did not report standardized resection rates and were therefore excluded from this analysis. Gross total resection (GTR) rates varied considerably across series, reflecting heterogeneity in tumor histology, patient populations, and definitions of surgical approach. Among studies employing the SCIT approach, GTR rates ranged from 45% to 95.8%, with the largest series reporting rates between 72% and 88.5% [1, 2, 31, 33, 39]. Studies primarily adopting the OITA approach reported more variable GTR rates, ranging from 58% to 96.3% [3, 5, 15]. Notably, the two comparative studies directly evaluating EOR between approaches consistently favored SCIT: Richards et al. reported a median EOR of approximately 97% for SCIT versus 60% for OITA (*p* = 0.04) [40], and Milisavljevic et al. reported GTR rates of 78% for SCIT versus 50% for OITA [8].

#### Comparison of SCIT vs OITA

 A statistical comparison of the complication profiles between the supracerebellar infratentorial (SCIT) and occipital interhemispheric transtentorial (OITA) approaches revealed several significant differences. Parinaud’s syndrome was significantly more frequent in the SCIT group (*p* = 0.000009), as were other complications such as death (*p* = 0.0059), CSF leak (*p* = 0.0029), meningitis (*p* = 0.000006), hemorrhage (*p* = 0.0033), and ataxia (*p* = 0.0042). On the other hand, homonymous hemianopia occurred more often following the OITA approach (*p* = 0.0011). Importantly, both Parinaud’s syndrome and homonymous hemianopia should be interpreted in the context of the selected surgical corridor and the preoperative tumor extension, as these approaches are often chosen for lesions with preferential inferior or superior growth patterns, respectively. Wound infections did not differ significantly between the two approaches (*p* = 0.574).

Table 3 summarizes the comparison of complication rates between the SCIT and OITA approaches.

**Table 3:** Comparison of complication rates between the supracerebellar infratentorial (SCIT) and occipital interhemispheric transtentorial (OITA) approaches

Complication	SCIT (n = 632)	OITA (n = 697)	p-value
Parinaud's syndrome	97 (15.35%)	35 (5.02%)	0.000009
Death	52 (8.23%)	11 (1.58%)	0.0059
Ataxia	36 (5.70%)	7 (1.00%)	0.0042
Meningitis	33 (5.22%)	3 (0.43%)	0.000006
CSF leak	25 (3.96%)	2 (0.29%)	0.0029
Hemorrhage	20 (3.16%)	2 (0.29%)	0.0033
Homonymous hemianopia	3 (0.47%)	38 (5.45%)	0.0011
Wound infection	9 (1.42%)	12 (1.72%)	0.574
Seizure	7 (1.11%)	8 (1.15%)	0.972
Pseudomeningocele	4 (0.63%)	2 (0.29%)	0.428
Air embolism	6 (0.95%)	1 (0.14%)	0.062

*CSF* cerebrospinal fluid

### Anatomical comparative study

Analysis of quadrant exposure across the two anatomical specimens showed differences in visualization patterns among the three approaches. In Head 1, SCIT and SCIT + T provided wider exposure than OITA in Q2 (80%, *p* < 0.05), while SCIT + T was associated with increased exposure in Q4 (26%, *p* < 0.0001). Exposure of Q1 was comparable among all three approaches (SCIT + T: 54%, SCIT: 53%, OITA: 44%), with no statistically significant differences (*p* = 0.759). OITA demonstrated greater visualization in Q3 (60%) compared with SCIT and SCIT + T (*p* = 0.0001).

In Head 2, infratentorial approaches (SCIT and SCIT + T) were associated with greater exposure in Q1 (81%, *p* < 0.05) and Q2 (60%, *p* < 0.001), while SCIT + T showed increased visualization of Q4 (14.3%, p < 0.0001). OITA again provided the greatest exposure in Q3 (63%, *p* < 0.001).

**Fig. 7:**
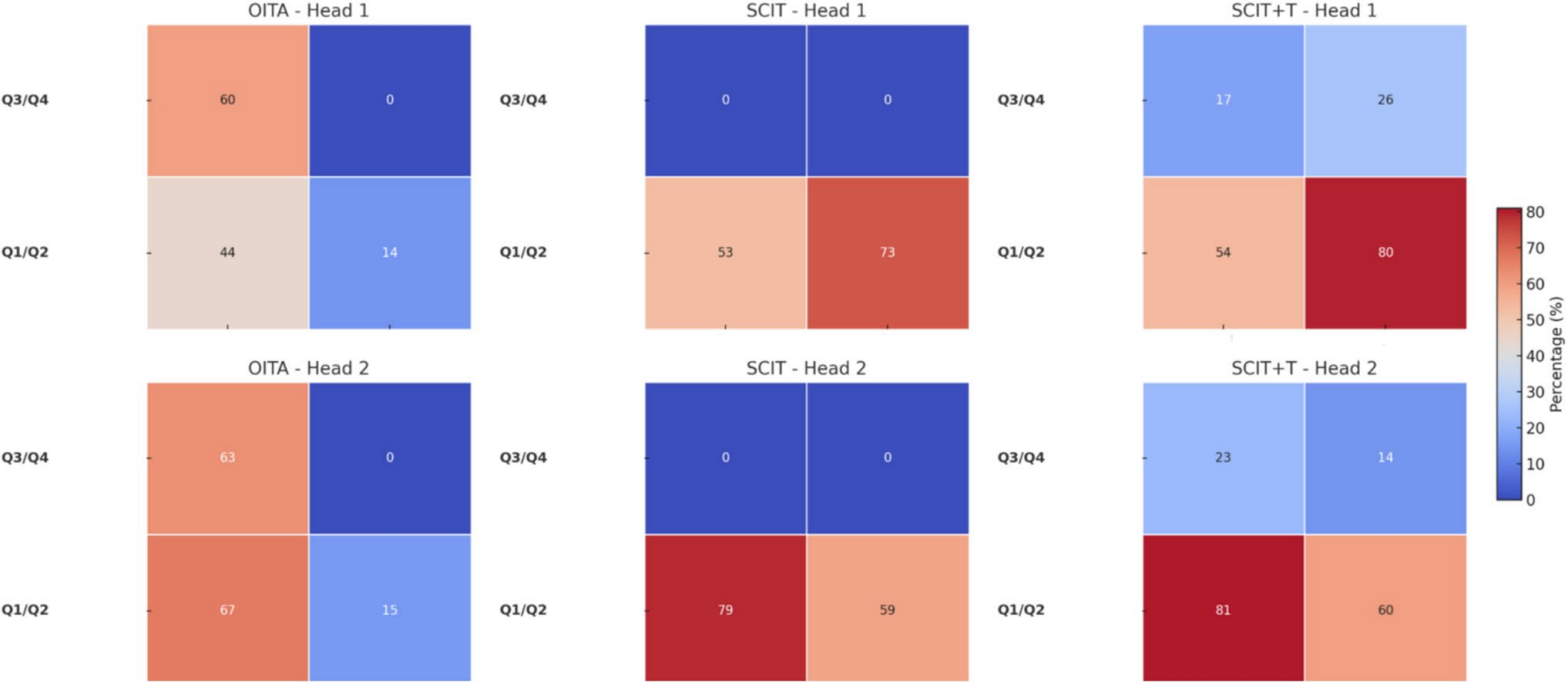
Heatmap representation of mean exposure volumes across surgical approaches and anatomical quadrants

Overall, these findings illustrate that OITA can provide exposure comparable to infratentorial approaches in the ipsilateral subtentorial quadrant (Q1), while the addition of a tentorial incision to the SCIT approach may facilitate visualization of supratentorial quadrants (Q3 and Q4), which are otherwise less accessible through the standard SCIT corridor. The HeatMap in Fig. 7 summarizes these exposure patterns.

## Discussion

The surgical management of pineal region tumors remains challenging due to their deep location and intimate relationship with critical neurovascular structures [18, 27, 41]. This systematic review highlights that the SCIT and OITA approaches represent the two main surgical corridors widely adopted in clinical practice [12, 13, 16, 33]. This review confirmed the lack of a definitive consensus on the optimal strategy, highlighting variability across surgeon experience, patient anatomical characteristics, and tumor morphology.

The analysis of complications from the literature revealed distinct profiles associated with each approach. Specifically, the SCIT approach demonstrated a significantly higher rate of cerebellar dysfunction, CSF leaks, meningitis, and Parinaud’s syndrome, likely reflecting its infratentorial trajectory and the need to sacrifice the bridging vein, which increases the risk of cerebellar venous infarction [6, 8, 14, 17]. Conversely, homonymous hemianopia was notably more frequent in patients treated via OITA, attributable to occipital lobe retraction and manipulation during the approach [3, 11, 29, 36].

The anatomical dissection and tumor simulation study provided additional clarity on the strengths and limitations of each approach across anatomical quadrants. Specifically, for the lower quadrants (Q1 and Q2), the infratentorial approaches (SCIT and SCIT + T) consistently provided superior visualization compared to the OITA approach. Nevertheless, OITA maintained relatively adequate exposure in the ipsilateral inferior quadrant (Q1), as shown in Head 1, where there was no statistical difference between the two. For the upper quadrant (Q3), OITA offered significantly superior visualization, a finding corroborated by previous anatomical studies [5, 11, 15, 40]. Interestingly, adding a tentorial incision to the SCIT approach (SCIT + T) markedly improved visibility of the supratentorial quadrants (Q3 and Q4), which are otherwise difficult to visualize adequately through the standard SCIT corridor. These findings align with the recent anatomical analyses by Milisavljevic et al. and Cinalli et al., further validating the advantages of a tailored approach incorporating a tentorial incision [8, 17].

The literature emphasizes several critical factors influencing the choice between SCIT and OITA, including the tumor’s anatomical characteristics, the angle of the tentorium, and, especially, the relationship with the venous system [19, 23, 24, 40]. Among these, evaluating the tumor’s relation with the ICVs and the VoG preoperatively is mandatory [23]. This tumor simulation study reflects this perspective: tumors anterior to the venous complex, as in Head 1, are more readily accessible via OITA, whereas tumors extending predominantly posteriorly to the veins, such as Head 2, favor SCIT as the optimal surgical corridor, aligning with recent anatomical and surgical studies [8, 16, 40, 47].

Another variable discussed in recent literature is the tentorial angle, with steeper angles traditionally considered unfavorable for SCIT [8, 16, 34]. The tentorial angle represents a key anatomical determinant when selecting a surgical corridor to the pineal region [17]. A steep tentorium may limit upward visualization and working angles during supracerebellar infratentorial approaches, whereas a flatter tentorial configuration can facilitate supratentorial access through occipital interhemispheric transtentorial routes [17]. Recent anatomical and clinical series have confirmed that tentorial inclination directly affects surgical exposure and working distance, reinforcing the concept that approach selection should be tailored to individual cranial anatomy rather than standardized across patients [8, 23, 40, 47]. However, this study suggests that with an adequate tentorial incision (SCIT + T), even steep angles can be managed effectively, expanding the utility and versatility of infratentorial corridors.

Beyond anatomical considerations, patient-specific factors should be integrated into surgical decision-making. Advanced age, cardiopulmonary comorbidities, or contraindications to the sitting or semi-sitting position may influence the feasibility of supracerebellar infratentorial approaches [1, 6, 14]. In these settings, supratentorial routes may offer a reasonable alternative [1].

Recently, fully endoscopic approaches to the pineal region have gained increasing attention [16, 48–51]. Although these techniques offer the advantage of a minimally invasive trajectory and potentially lower morbidity, they present limitations in large or highly vascularized lesions [49]. As shown in the literature, a combined microscopic-endoscopic approach could be a valid option, proving the strengths of both techniques: the microscope provides the necessary precision and control during tumor resection, whereas the endoscope addresses the inherent "blind spots" of each approach, facilitating inspection of hidden anatomical recesses, thus potentially reducing residual tumor and complication rates [16, 47]. Moreover, high-definition exoscopic systems have recently been introduced as adjuncts or alternatives to operative microscopes, providing enhanced 3D visualization and improved surgeon comfort during deep surgical approaches [52, 53].

Therefore, while both SCIT and OITA present well-defined strengths and limitations, extensively described in the literature, the choice of the optimal surgical approach should always be tailored to the individual patient's anatomy and tumor characteristics, especially the relationship with the deep venous system [8, 47]. It should also be acknowledged that surgeon experience, institutional preference, and familiarity with specific anatomical corridors are legitimate and clinically meaningful determinants of approach selection that are intrinsically difficult to capture in a retrospective comparative analysis. These individual-level factors inevitably introduce a selection bias in the interpretation of approach-specific complication rates, and their influence should be considered when interpreting the statistical comparisons presented in this study. Taken together, SCIT and OITA represent complementary rather than competing approaches. SCIT offers a direct infratentorial trajectory with excellent exposure of the inferior pineal region (Q1 + Q2), at the cost of potential venous and cerebellar-related morbidity. Conversely, OITA provides superior visualization of the superior quadrants (Q3 or Q4) and of lesions extending above the deep venous system, while exposing patients to a higher risk of visual field deficits associated with occipital lobe manipulation [47]. However, it should be mentioned that for large tumors with predominant anterior extension into the third ventricle, or lesions above the Herophilus-Galenic line, both SCIT and OITA may offer suboptimal exposure, and the interhemispheric transcallosal approach should be considered [7, 17].

In conclusion, this article may help young consultants in approaching decision-making for pineal region tumors, and for this reason, we have proposed a practical Decision-Making Summary for Approach Selection (Table 4). Dividing a tumor into four quadrants and considering its extent within them can better inform the approach; however, this framework should always be integrated with a careful assessment of venous anatomy, tentorial configuration, and patient-specific factors.

**Table 4:** Practical decision-making framework for surgical approach selection in pineal region tumors

Key Variable	Favors SCIT (± tentorial incision)	Favors OITA (or transcallosal)
Tumor position relative to the deep venous system	Posterior to ICVs/VoG	Anterior to ICVs/VoG
Predominant tumor extension	Inferior quadrants Q1, Q2 (infratentorial)	Superior ipsilateral Q3 (supratentorial) or anterior 3rd ventricle
Tentorial angle	Flat-to-intermediate (SCIT + T for steep angles)	Very steep (OITA may offer better supratentorial exposure)
Tumor size	Small-to-medium, posterior, capsule-intact	Large with anterior/superior extension → consider transcallosal
Patient factors	No contraindications to semi-sitting; good cardiopulmonary reserve	Cardiopulmonary comorbidities; contraindication to semi-sitting
Adjunct techniques	Tentorial incision (SCIT + T); endoscope for blind spots	Endoscope for infratentorial recesses; combined SCIT + OITA for complex cases

*ICVs* internal cerebral veins, *VoG* vein of Galen, *SCIT + T* SCIT with tentorial incision

### Strengths and limitations of the study

This study offers significant methodological strengths, including a comprehensive dual approach that combines a systematic literature review with anatomical cadaveric dissections. Combining these methodologies provides robust validation of findings by integrating clinical and anatomical insights. Furthermore, employing the tumor simulation technique with a balloon filled with contrast media notably enhances the anatomical study, offering realistic visualization and analysis of surgical exposure and tumor-neurovascular relationships, thereby improving the clinical relevance and applicability of the anatomical results.

Despite these strengths, several limitations should be acknowledged. Firstly, the anatomical study involved only two cadaveric specimens, limiting the generalizability of the anatomical findings. Although a specimen with a less steep tentorial angle (Specimen n.2) was included for comparative purposes, the availability of a specimen with an even flatter tentorium might have further refined the anatomical analysis, whose results should be interpreted with caution and not as evidence of definitive superiority of one approach over another. This was a preliminary study, and in the future, a larger number of specimens would be necessary to assess anatomical variability, particularly in venous configurations and tentorial morphology. Additionally, this study did not include fully endoscopic surgical series, which may have potentially limited insights into the efficacy and applicability of purely endoscopic approaches. Another limitation of the present study is that it does not include the full spectrum of microsurgical variants used to access the pineal region. Furthermore, the tumor simulation model, based on a water balloon tumor (WBT), replicates the behavior of a round, expansile, capsule-intact lesion that is pathobiologically more representative of benign tumors or small- to medium-sized malignant lesions with intracapsular growth. Large malignant pineal tumors frequently destroy their capsule and invade or encase surrounding neurovascular structures; such scenarios cannot be replicated by the WBT simulation, and the conclusions drawn may not be directly applicable to highly infiltrative lesions. Finally, EOR data were available only in a subset of the included studies and in non-uniform formats, precluding a formal meta-analytic comparison of resection rates between SCIT and OITA. In particular, several paramedian and modified supracerebellar infratentorial approaches, as extensively described in the literature, may offer different working angles, venous management strategies, and exposure characteristics compared with the midline corridors analyzed in this study. For example, the anatomical and simulation analyses might not be directly applicable to anteriorly projecting tumors extending into the third ventricle, for which other surgical approaches, such as the interhemispheric transcallosal approach, could offer superior outcomes. These variations were not specifically modeled in the cadaveric simulations and were therefore excluded from the quantitative assessment. Moreover, the wide variability in patient populations, particularly regarding age distribution across pediatric and adult cohorts, further reduced homogeneity.

## Conclusion

The surgical management of pineal region tumors remains complex due to the region’s deep anatomical location and the critical neurovascular structures surrounding it. This comprehensive analysis indicates that both SCIT and OITA approaches offer distinct advantages and limitations. SCIT provides superior visualization of lower quadrants but is associated with higher rates of cerebellar complications, Parinaud’s syndrome, cerebrospinal fluid leaks, and venous infarction. In contrast, OITA provides better visualization of the superior ipsilateral quadrant but carries an increased risk of homonymous hemianopia due to manipulation of the occipital lobe. The anatomical dissections, combined with the tumor simulation, further demonstrate that adding a tentorial incision to SCIT (SCIT + T) enhances visualization of the upper quadrants, making it a versatile approach even in cases of steep tentorial angles.

In conclusion, the present study does not aim to define a universally superior surgical approach. Rather, it suggests that the “best” surgical approach for pineal region tumors is the one that best fits the individual anatomical configuration, tumor extension, venous relationships, and patient-specific factors. When these elements are considered together, both supracerebellar infratentorial and occipital interhemispheric transtentorial corridors can be safely and effectively applied within a tailored, patient-centered surgical strategy.

## Data Availability

The data supporting the findings of this study are available from the corresponding author upon reasonable request.
